# Assisting Movement Training and Execution With Visual and Haptic Feedback

**DOI:** 10.3389/fnbot.2018.00024

**Published:** 2018-05-29

**Authors:** Marco Ewerton, David Rother, Jakob Weimar, Gerrit Kollegger, Josef Wiemeyer, Jan Peters, Guilherme Maeda

**Affiliations:** ^1^Intelligent Autonomous Systems Group, Department of Computer Science, Technische Universität Darmstadt, Darmstadt, Germany; ^2^Institute of Sport Science, Technische Universität Darmstadt, Darmstadt, Germany; ^3^Max Planck Institute for Intelligent Systems, Tübingen, Germany; ^4^ATR Computational Neuroscience Labs, Department of Brain-Robot Interface, Kyoto, Japan

**Keywords:** shared autonomy, HRI, movement primitives, reinforcement learning, policy search, cooperation, robotics, interaction

## Abstract

In the practice of motor skills in general, errors in the execution of movements may go unnoticed when a human instructor is not available. In this case, a computer system or robotic device able to detect movement errors and propose corrections would be of great help. This paper addresses the problem of how to detect such execution errors and how to provide feedback to the human to correct his/her motor skill using a general, principled methodology based on imitation learning. The core idea is to compare the observed skill with a probabilistic model learned from expert demonstrations. The intensity of the feedback is regulated by the likelihood of the model given the observed skill. Based on demonstrations, our system can, for example, detect errors in the writing of characters with multiple strokes. Moreover, by using a haptic device, the Haption Virtuose 6D, we demonstrate a method to generate haptic feedback based on a distribution over trajectories, which could be used as an auxiliary means of communication between an instructor and an apprentice. Additionally, given a performance measurement, the haptic device can help the human discover and perform better movements to solve a given task. In this case, the human first tries a few times to solve the task without assistance. Our framework, in turn, uses a reinforcement learning algorithm to compute haptic feedback, which guides the human toward better solutions.

## 1. Introduction

In the absence of an instructor, errors in the execution of movements by a person trying to learn a new motor skill, such as calligraphy, for example, may go unnoticed. To counter this problem, we propose recording demonstrations of a motor skill provided by an instructor and processing them such that someone practicing that motor skill in the absence of the instructor can have the correctness of his/her trials automatically assessed and receive feedback based on the demonstrations.

More precisely, our system aligns demonstrated trajectories in space and time and computes a probability distribution over them. Often, demonstrations may have been executed at different speeds. In order to extract the underlying shape of the movement from multiple trajectories, it is thus necessary to time-align these trajectories. In some cases, such as writing characters, the scale and the absolute position of the movements are not as relevant as their shape, justifying the necessity of addressing space-alignment in our framework as well.

When a new trajectory is executed, our system aligns the observations in space and time with the post-processed demonstrations and computes the probability of each of the positions of this new trajectory under the distribution over the demonstrations. The computed probabilities provide a way of assessing the correctness of each position of the new trajectory.

**Figure 1:**
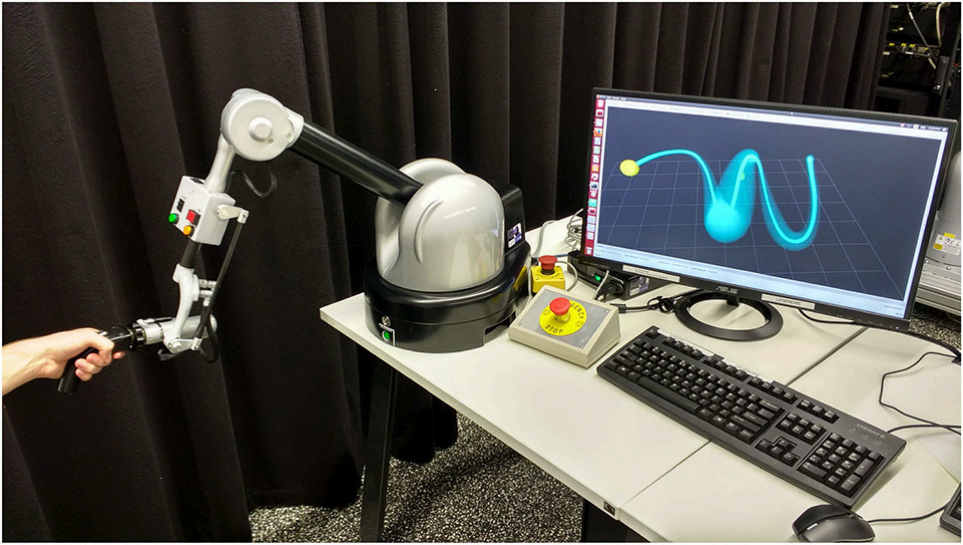
Human manipulating a haptic device, the Haption Virtuose 6D. In our experiments, the haptic device assists the movements of the human by providing force feedback which is inversely proportional to the standard deviation of a distribution over trajectories (example is shown on the computer screen).

Based on this assessment, our system can generate visual or haptic feedback. We demonstrate the generation of visual feedback with the task of assisting the practice of writing Japanese characters on a monitor with a computer mouse. The generation of haptic feedback is demonstrated in an experiment with a haptic device, the Haption Virtuose 6D (see Figure 1). Our system gives haptic feedback to the user in the form of forces that constrain his/her movements when manipulating the haptic device, which can be seen as a form of guiding virtual fixtures (Rosenberg, 1992). The produced force is perpendicular to the mean trajectory of the distribution and its intensity is inversely proportional to the standard deviation along the distribution, as detailed in section 4.

There are situations where the provided demonstrations do not contain truly expert skills, and thus cannot successfully be used to build the probabilistic model. Nevertheless, it may be possible to define performance measurements accounting for certain objectives. Examples of such a situation could be found in a teleoperation task where the user perception and motor capabilities do not enable him/her to succeed. Such a task could be for instance telemanipulating a robot arm to move an object from a start position to an end position while avoiding obstacles. In such a task, a user can easily hit obstacles or fail to reach objects of interest. However, it may be possible to define performance measurements based on the positions of objects in the environment of the teleoperated robot. These positions could be computed from information provided by sensors in that environment. The framework presented in this paper deals with these situations by applying reinforcement learning to adapt the original distribution over trajectories. The adapted distribution is then used to guide the user toward a better solution to the task.

In general, the problem of finding a distribution over trajectories that avoid obstacles and pass through positions of interest involves multiple optimization subproblems. Tuning the hyperparameters of the reward function to satisfy all the objectives may be time-consuming and may not produce the desired results. For this reason, our proposed framework includes a novel reinforcement learning algorithm that makes use of a parametric representation of trajectories and identifies how relevant each policy parameter is to each of the objectives of the task. By identifying how relevant each policy parameter is to each objective, it is possible to achieve effective policies with simpler reward functions, one for each objective, instead of a single reward function with different user-defined weights for each objective. Moreover, it is possible to optimize each objective sequentially, exploring different values of the parameters that matter for that objective and preserving the uncertainty about the other parameters.

In summary, this paper presents a new framework to assist humans in training and executing movements by providing visual and haptic feedback to the user. This feedback can be given based on a probability distribution over expert demonstrations or based on an optimized distribution learned from a few non-expert demonstrations and performance criteria. By including methods for time and space-alignment of trajectories, this framework can potentially be applied to a large range of motor skills as long as the shape of the movement is critical, not its speed. In this work, our framework has been applied to the learning of Japanese characters and to teleoperation. As a secondary contribution, this paper presents a novel reinforcement learning algorithm for problems involving multiple objectives, which are often encountered in teleoperation scenarios.

## 2. Related work

This section primarily describes related work on techniques to assess the correctness of human motion and provide feedback to the user. It briefly introduces related work on the required components used for modeling the human demonstrations.

### 2.1. Human motion assessment and feedback to the user

With similar goals as in our work, Solis et al. (2002) presented a method to teach users how to write characters using a haptic interface. In their method, characters are modeled with Hidden Markov Models (HMMs) with discrete hidden states and discrete observations. The system recognizes online what character the user intends to write and applies a proportional derivative (PD) controller with fixed gains to restrict the user to move along the trajectory that corresponds to the recognized character. Differently, in our work, the gains of the haptic device are adapted as a function of the user's deviation with respect to the model learned from expert demonstrations or through reinforcement learning. Adaptive gains allow for practicing motor skills with multiple correct possibilities of execution, in case there is not a single correct trajectory. Also, it allows for regulating the stiffness of the robot to impose different levels of precision at different parts of the movement.

Parisi et al. (2016) proposed a “multilayer learning architecture with incremental self-organizing networks” to give the user real-time visual feedback during the execution of movements, e.g., powerlifting exercises. In our work, we have not addressed real-time visual feedback so far, although we do address real-time haptic feedback. On the other hand, our framework can deal with movements with different absolute positions and scales when producing visual feedback. By disabling this preprocessing, it would be possible to generate real-time visual feedback as well.

Kowsar et al. (2016) presented a workflow to detect anomalies in weight training exercises. In their work, movement repetitions are segmented based on the acceleration along an axis in space. A probability distribution over a number of time-aligned repetitions is built. Then, based on this distribution, movement segments can be deemed correct or incorrect. Our approach focuses rather on correcting movements with respect to their shape or position in space, not on correcting acceleration patterns.

A variable impedance controller based on an estimation of the stiffness of the human arm was proposed by Tsumugiwa et al. (2002). This controller enabled a robot to assist humans in calligraphic tasks. In the cited work, the tracked trajectories were not learned from demonstrations.

Our work is in line with approaches that aim to assist learning with demonstrations. Raiola et al. (2015), for instance, used probabilistic virtual guides learned from demonstrations to help humans manipulate a robot arm. In another related work, Soh and Demiris (2015) presented a system that learns from demonstrations how to assist humans using a smart wheelchair.

Visual, auditory and haptic feedback modalities have been successfully used for motor learning in the fields of sport and rehabilitation (Sigrist et al., 2013). Our method to provide visual feedback to the user, detailed in section 3.4, is, for instance, similar in principle to bandwidth feedback. This sort of feedback means that the user only receives feedback when the movement error exceeds a certain threshold and it has been shown to be effective in rehabilitation (Timmermans et al., 2009). The work here presented relates and can potentially complement previous research on bandwidth feedback in the sense that our threshold is not constant, but depends on a probability distribution over trajectories. Our approach may find applications in tasks where it is desirable to give the user more freedom of movement around a certain position and less freedom around a different position or where multiple variations of movements are considered correct.

Ernst and Banks (2002) have demonstrated that maximum-likelihood estimation describes the way humans combine visual and haptic perception. The estimation of a certain environmental property that results from the combination of visual and haptic stimuli presents lower variance than estimations based only on visual or haptic stimuli. When the visual stimulus is noise-free, users tend to rely more on vision to perform their estimation. On the other hand, when the visual stimulus is noisy, users tend to rely more on haptics. Therefore, users may profit from multimodal feedback to learn a new motor skill. In our experimental section, we provide haptic feedback to users to help them perform a teleoperation task in a virtual environment. The findings in Ernst and Banks (2002) indicate that haptic feedback also helps users perceive some aspects of the task that they could not perceive only from visual stimuli, which could help them learn how to better solve the task without assistance next time. The usefulness of haptic feedback to learn motor skills is also demonstrated in Kümmel et al. (2014), where robotic haptic guidance has been shown to induce long-lasting changes in golf swing movements. The work here presented offers an algorithmic solution to the acquisition of policies and control of a robotic device that could be applied to help humans learn and retain motor skills.

In contrast to most of the work on haptic feedback for human motor learning, our method modulates the stiffness of the haptic device according to demonstrations and uses reinforcement learning to improve upon the demonstrated movements. Those features may be interesting as a means of communication between an expert and an apprentice or patient and to enable improvement of initial demonstrations.

### 2.2. Learning and adapting models from demonstrations

An essential component of this work is to construct a model from expert demonstrations, which is then queried at runtime to evaluate the performance of the user. One recurrent issue when building models from demonstration is the problem of handling the variability of phases (i.e., the speed of the execution) of different movements. Listgarten et al. (2004) proposed the Continuous Profile Model (CPM), which can align multiple continuous time series. It assumes that each continuous time series is a non-uniformly subsampled, noisy and locally rescaled version of a single latent trace. The model is similar to a Hidden Markov Model (HMM). The hidden states encode the corresponding time step of the latent trace and a rescaling factor. The CPM has been successfully applied to align speech data and data sets from an experimental biology laboratory.

Coates et al. (2008) augmented the model of Listgarten et al. (2004) by additionally learning the dynamics of the controlled system in the vicinity of the intended trajectory. With this modification, their model generates an ideal trajectory that not only is similar to the demonstrations but also obeys the system's dynamics. Moreover, differently from Listgarten et al. (2004), their algorithm to time-align the demonstrations and to determine an ideal trajectory relies both on an EM algorithm and on Dynamic Time Warping (Sakoe and Chiba, 1978). With this approach, they were able to achieve autonomous helicopter aerobatics after training with suboptimal human expert demonstrations.

The same method was used by Van Den Berg et al. (2010) to extract an ideal trajectory from multiple demonstrations. The demonstrations were, in this case, movements of a surgical robot operated by a human expert.

Similarly to Coates et al. (2008) and Van Den Berg et al. (2010), our system uses Dynamic Time Warping (DTW) to time-align trajectories. While DTW usually aligns pairs of temporal sequences, in section 3.2 we present a solution for aligning multiple trajectories. An alternative solution was presented by Sanguansat (2012), however, it suffers from scalability issues because distances need to be computed between every point of every temporal sequence.

Differences in the scale and shape of movements must also be addressed to account for the variability in human demonstrations. In practice, for tasks such as writing, we want our system to be invariant to the scale of the movements of different demonstrations. The analysis of the difference between shapes is usually addressed by Procrustes Analysis (Goodall, 1991). The output of this analysis is the affine transformation that maps one of the inputs to best match the other input, while the residual is quantified as the effective distance (deformation) between the shapes. As the analysis consists of computing such transformations in relation to the centroid, Procrustes Analysis provides a global, average assessment and has found applications in tasks of trajectory and transfer learning (Bocsi et al., 2013; Makondo et al., 2015; Holladay and Srinivasa, 2016) and manipulation (Collet et al., 2009). While this seems the most natural solution to our problem of aligning shapes, we noticed that it is not suitable for detecting anomalies. In fact, in the writing task, we are interested in finding the “outliers” that can be indicated to the human as erroneous strokes. However, Procrustes Analysis aligns the shapes globally such that the positions of the centroids are inappropriately biased toward such outliers. In sections 3.1.1 and 3.1.2 we describe our own alignment method that is suited for detecting particular errors with the introduction of a few heuristics.

## 3. Processing demonstrations and assessing the correctness of observed trajectories

Assuming the availability of expert demonstrations, the workflow of our proposed method is the following: First, the expert demonstrations are aligned in space and time and a probability distribution over these demonstrations is computed. Afterward, a user tries to perform the motor task. The movements of the user are also aligned in space and time with the demonstrations. Based on the probability distribution over the demonstrations, our system highlights which parts of the user's movements need improvement. A way of translating a distribution over trajectories into haptic feedback is presented later in section 4. A novel reinforcement learning algorithm is presented in section 5. The algorithm attempts to improve the movements of the user according to certain performance criteria, even when the initial demonstrations are considered suboptimal under the same criteria.

### 3.1. Rescaling and repositioning

In assessing the correctness of individual executions of a motor skill, it is often not important what the absolute position of the sequence of movements is, e.g., in weightlifting or gymnastics. In some situations, it is also not of crucial importance what the scale of the movements is as long as they keep their relative proportions, e.g., in drawing or calligraphy. Therefore, our system rescales all trajectories, both the ones demonstrated by a human expert and the ones performed by a user practicing a motor skill. Moreover, all trajectories are repositioned in such a way that the first position of the reference stroke is at the origin of the coordinate system. In practice, each stroke composing a motor skill is used once as the reference for rescaling and repositioning. For each reference stroke, a different score and visual feedback are computed. The best score and the respective feedback are presented to the user. This procedure enables our algorithm to present meaningful feedback to the user regardless the location of his/her errors. In this section, our method for rescaling and repositioning is explained for two dimensions (*x* and *y*) and exemplified with the task of writing Japanese characters. This method can nevertheless be extended in a straightforward manner for more than two dimensions.

#### 3.1.1. Rescaling

First, the system computes

(1) $$\Delta x_{\text{ref}}=\underset{t}{\max}\;x_{\text{ref}}\left(t\right)-\underset{t}{\min}\;x_{\text{ref}}\left(t\right),$$

(2) $$\Delta y_{\text{ref}}=\underset{t}{\max}\;y_{\text{ref}}\left(t\right)-\underset{t}{\min}\;y_{\text{ref}}\left(t\right),$$

where *t* indexes each time step, $\underset{t}{\max}x_{\text{ref}}\left(t\right)$ is the maximum *x* coordinate of the reference stroke, $\underset{t}{\min}x_{\text{ref}}\left(t\right)$ is the minimum *x* coordinate of the reference stroke, and similarly for $\underset{t}{\max}y_{\text{ref}}\left(t\right)$ and $\underset{t}{\min}y_{\text{ref}}\left(t\right)$.

Subsequently, a rescaling factor α is given by

(3) $$\alpha =\{\begin{array}{ll} \frac{1}{\Delta x_{\text{ref}}} & \text{if }\Delta x_{\text{ref}}\geq \Delta y_{\text{ref}},\\ \frac{1}{\Delta y_{\text{ref}}} & \text{otherwise}. \end{array}$$

**Figure 2:**
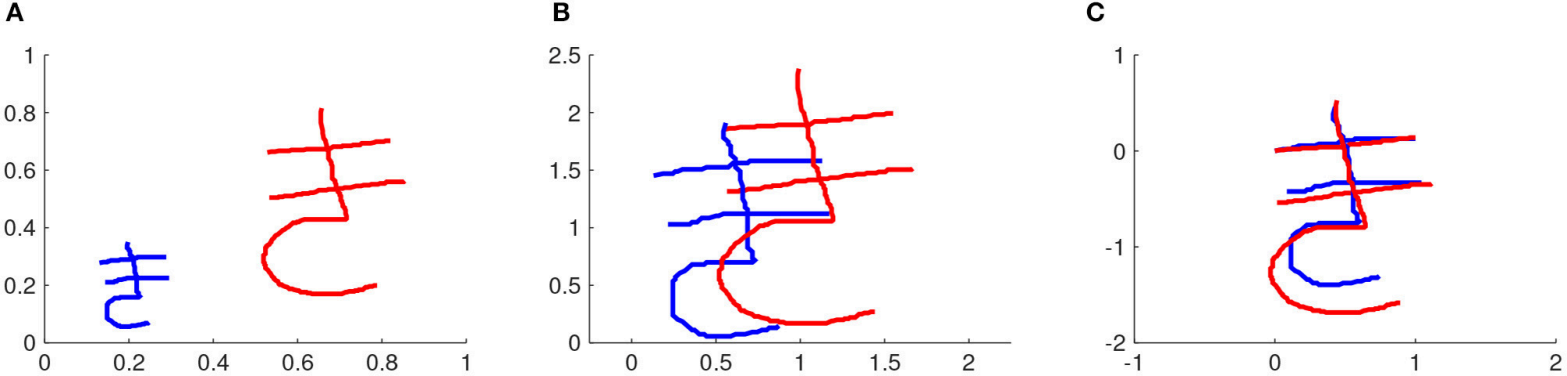
Rescaling and repositioning different executions of a motor skill. In this example, the motor skill is writing a Japanese character. **(A)** Two demonstrations of a Japanese character. **(B)** After rescaling both characters. **(C)** After repositioning the characters such that the first position of the first stroke is (*x* = 0, *y* = 0). The first stroke is the reference stroke in this case.

The characters are written on a square window with side equal to 1. The rescaling factor α expresses the ratio between the constant 1 and the width Δ*x*_ref_ or height Δ*y*_ref_ of the reference stroke. If Δ*x*_ref_ ≥ Δ*y*_ref_, the width is used to compute α. Otherwise, the height is used. Some strokes are much larger in width than in height or vice versa. Therefore, this way of computing the rescaling factor selects the width or the height of the reference stroke according to which one will lead to the smallest amount of rescaling. For example, the characters depicted in Figure 2A will be rescaled according to the width of the first stroke of each of them respectively, resulting in characters whose first stroke has width equal to 1.

The rescaling factor can also be written as

(4) $$\alpha =\frac{x_{i,\text{rescaled}}\left(t\right)-\underset{\left\{j,k\right\}}{\min}\;x_{j}\left(k\right)}{x_{i}\left(t\right)-\underset{\left\{j,k\right\}}{\min}\;x_{j}\left(k\right)}=\frac{y_{i,\text{rescaled}}\left(t\right)-\underset{\left\{j,k\right\}}{\min}\;y_{j}\left(k\right)}{y_{i}\left(t\right)-\underset{\left\{j,k\right\}}{\min}\;y_{j}\left(k\right)},$$

where both *t* and *k* are time step indexes, while the indexes *i* and *j* represent the strokes of a character. Here, $x_{i,\text{rescaled}}\left(t\right)-\underset{\left\{j,k\right\}}{\min}x_{j}\left(k\right)$ is the difference between the *x* coordinate at time step *t* of stroke *i* after rescaling and the minimum *x* coordinate of the character. The term $x_{i}\left(t\right)-\underset{\left\{j,k\right\}}{\min}x_{j}\left(k\right)$ represents the corresponding difference before rescaling. Equation (4) also includes similar terms for the *y* coordinates. Therefore, after rescaling, the difference between the *x* coordinate of the position at time step *t* and the minimum *x* coordinate is α times this difference before rescaling, and similarly for the *y* coordinate. Thus this rescaling keeps the proportion between the width and the height of the character.

Rearranging the terms of Equation (4) leads to

(5) $$x_{i,\text{rescaled}}\left(t\right)=\underset{\left\{j,k\right\}}{\min}\;x_{j}\left(k\right)+\left(x_{i}\left(t\right)-\underset{\left\{j,k\right\}}{\min}\;x_{j}\left(k\right)\right)\alpha ,$$

(6) $$y_{i,\text{rescaled}}\left(t\right)=\underset{\left\{j,k\right\}}{\min}\;y_{j}\left(k\right)+\left(y_{i}\left(t\right)-\underset{\left\{j,k\right\}}{\min}\;y_{j}\left(k\right)\right)\alpha ,$$

which is how the coordinates of the rescaled version of a character are computed. Figure 2A shows two demonstrations of the same character and Figure 2B shows the result of rescaling these characters.

#### 3.1.2. Repositioning

In order to reposition a character such that the first position of the reference stroke is (*x* = 0, *y* = 0), our system simply computes

(7) $$x_{i,\text{repositioned}}\left(t\right)=x_{i}\left(t\right)-x_{\text{ref}}\left(t=1\right),$$

(8) $$y_{i,\text{repositioned}}\left(t\right)=y_{i}\left(t\right)-y_{\text{ref}}\left(t=1\right),$$

where *x*_*i*_(*t*) and *y*_*i*_(*t*) are the original coordinates of stroke *i* at time step *t*, *x*_*i*,repositioned_(*t*) and *y*_*i*,repositioned_(*t*) are the coordinates of stroke *i* at time step *t* of the character after repositioning, *x*_ref_(*t* = 1) and *y*_ref_(*t* = 1) are the coordinates of the reference stroke at the first time step. Figure 2C shows two demonstrations of the same character after rescaling and repositioning.

### 3.2. Time alignment

The time alignment of all the demonstrations and of the user's movements is achieved in our system by using Dynamic Time Warping (Sakoe and Chiba, 1978). Each stroke of an execution of a motor skill is time-aligned with respect to the corresponding stroke of other executions of that same motor skill.

Suppose two corresponding strokes need to be time-aligned. Let us represent these strokes by ***τ***_1_ and ***τ***_2_, which are sequences of Cartesian coordinates from time step *t* = 1 until time step *t* = *T*_1_ and *t* = *T*_2_, respectively. Here, *T*_1_ and *T*_2_ represent the last time step of ***τ***_1_ and ***τ***_2_, respectively.

First, the Euclidean distance ***D***(*i, j*) between position at *t* = *i* of ***τ*****_1_** and position at *t* = *j* of ***τ*****_2_** is computed for all time steps of both strokes, i.e.,

(9) $$\begin{array}{l} D\left(i,j\right)=\|\tau_{1}\left(i\right)-\tau_{2}\left(j\right)\|,\\ \forall i\in \left\{1,2,\cdots ,T_{1}\right\},\forall j\in \left\{1,2,\cdots ,T_{2}\right\}. \end{array}$$

Subsequently, assuming that the first position of ***τ***_1_ corresponds to the first position of ***τ***_2_, the accumulated cost ***A***(*i, j*) of associating ***τ***_1_(*i*) with ***τ***_2_(*j*) is computed according to

(10) A(1,1)=D(1,1),

(11) A(i,1)=D(i,1)+A(i−1,1),

(12) A(1,j)=D(1,j)+A(1,j−1),

(13) A(i,j)=D(i,j)+min{A(i−1,j), A(i−1,j−1), A(i,j−1)}.

Once the matrix of accumulated costs ***A*** has been determined, a path ***p*** can be computed that indicates how each trajectory should progress in time such that the minimum total cost is achieved. This path is computed backward in time in a dynamic programming fashion, as detailed in Algorithm 1.

**Algorithm 1:**
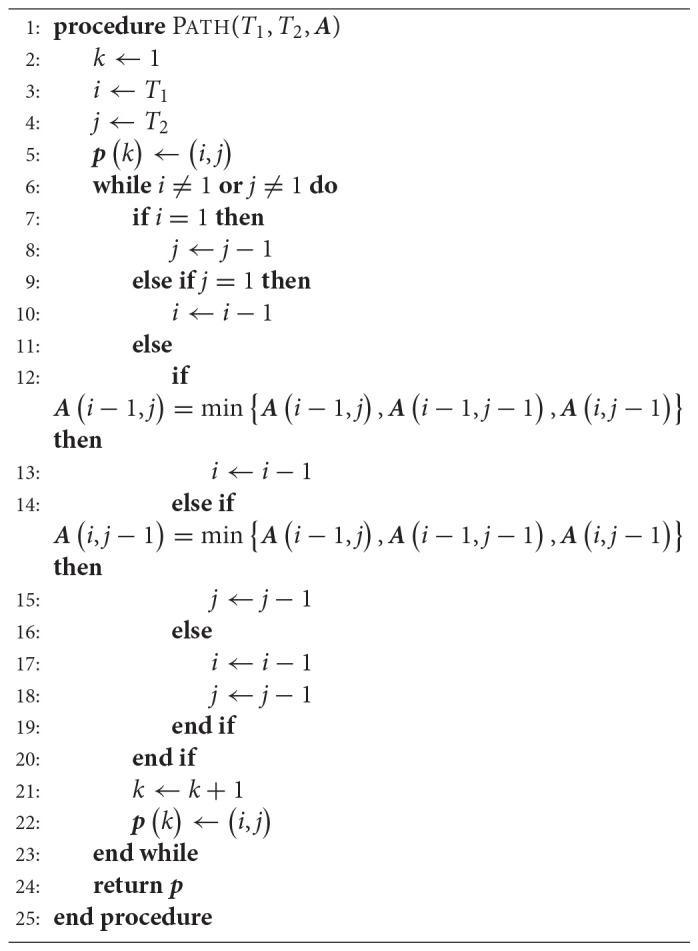
Path Search

The time warped versions of trajectories ***τ***_1_ and ***τ***_2_, denoted by $\tau_{1}^{\prime }$ and $\tau_{2}^{\prime }$, are computed with Algorithm 2.

**Algorithm 2:**
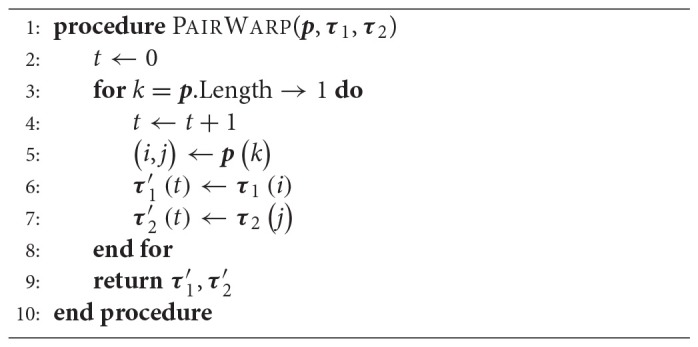
Warping for a Pair of Trajectories

**Figure 3:**
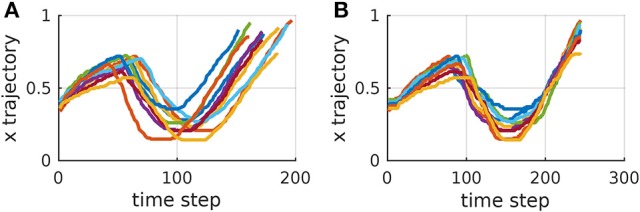
*x* trajectories of corresponding strokes of multiple instances of a Japanese character. **(A)** Before time alignment. **(B)** After time alignment using DTW and our extension to deal with multiple trajectories.

Algorithms 1 and 2 represent a common form of DTW which aligns pairs of temporal sequences. Algorithm 3 shows our proposed extension of DTW for time-aligning multiple temporal sequences. It works as follows: Trajectories ***τ***_1_ and ***τ***_2_ are time-aligned with DTW, resulting in $\tau_{1}^{\prime }$ and $\tau_{2}^{\prime }$. Then $\tau_{2}^{\prime }$ and ***τ***_3_ are time-aligned. Subsequently, the same warping applied to $\tau_{2}^{\prime }$ is also applied to $\tau_{1}^{\prime }$. The algorithm proceeds like that until ***τ***_*n*_, always warping previous trajectories as well. For *n* trajectories, DTW needs to be computed *n* − 1 times and the computation of the distance matrix ***D*** remains the same as in the original DTW. Figure 3 exemplifies the time-alignment of multiple trajectories.

**Algorithm 3:**
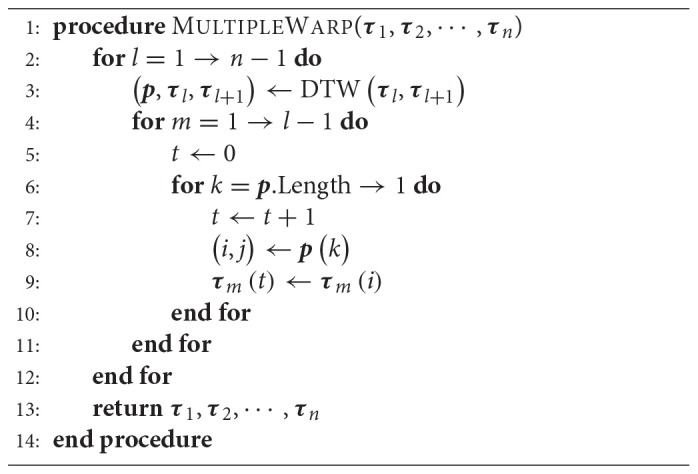
Warping for Multiple Trajectories

### 3.3. Distribution over trajectories

In order to create a distribution over trajectories, we use the framework of Probabilistic Movement Primitives (Paraschos et al., 2013). Probabilistic Movement Primitives (ProMPs) allow for representing each trajectory with a relatively small number of parameters. A distribution over trajectories can then be computed by integrating out those parameters.

More precisely, in this framework, each trajectory ***τ*** with a certain duration *T* is approximated by a weighted sum of *N* normalized Gaussian basis functions evenly distributed along the time axis. This approximation can be represented by

(14) τ= Ψw+ ϵ,

where ***w*** is a weight vector, ***ϵ*** is a zero-mean i.i.d. Gaussian noise, i.e., $\epsilon \sim N\left(0,\sigma^{2}I_{T\times T}\right)$, and

(15) $$\begin{array}{l} \Psi =\left[\begin{array}{cccc} \psi_{1}\left(1\right) & \psi_{2}\left(1\right) & \cdots & \psi_{N}\left(1\right)\\ \psi_{1}\left(2\right) & \psi_{2}\left(2\right) & \cdots & \psi_{N}\left(2\right)\\ \vdots & \vdots & \ddots & \vdots \\ \psi_{1}\left(T\right) & \psi_{2}\left(T\right) & \cdots & \psi_{N}\left(T\right) \end{array}\right]. \end{array}$$

A term ψ_*i*_(*t*) in this matrix represents the normalized Gaussian basis function with index *i* evaluated at time step *t*.

Given a trajectory ***τ***, a pre-defined matrix of basis functions **Ψ** and a regularizing factor λ, the weight vector ***w*** can be computed with linear ridge regression as follows:

(16) $$w={\left(\Psi^{T}\Psi +\text{λ}I_{N\times N}\right)}^{-1}\Psi^{T}\tau .$$

Once the weight vectors ***w*** corresponding to a set of trajectories ***τ*** have been computed, a Gaussian distribution $N\left(\mu_{w},\Sigma_{w}\right)$ over these vectors is determined using maximum likelihood estimation. The distribution over trajectories ***τ*** can be expressed as the marginal distribution

(17) p(τ)=∫p(τ|w)p(w)dw,

where $p\left(w\right)=N\left(w|\mu_{w},\Sigma_{w}\right).$ Assuming that a Gaussian is a good approximation for the distribution over ***w***, this integral can be solved in closed-form, yielding

(18) $$p\left(\tau \right)=N\left(\tau |\mu_{\tau },\;\Sigma_{\tau }\right),$$

with

(19) $$\begin{array}{l} \mu_{\tau }=\Psi \mu_{w},\\ \Sigma_{\tau }=\sigma^{2}I_{T\times T}+\Psi \Sigma_{w}\Psi^{T}. \end{array}$$

To deal with not only one stroke and a single degree of freedom (DoF) but with multiple strokes and multiple DoFs, one can think of ***τ*** as a concatenation of trajectories. The matrix **Ψ** becomes, in this case, a block diagonal matrix and ***w*** a concatenation of weight vectors. For further details about this formulation, the interested reader is referred to our previous work (Maeda et al., 2016) in which ProMPs were used to coordinate the movements of a human and a robot in collaborative scenarios.

The variance σ^2^ defining the Gaussian noise ***ϵ*** determines how sensitive our system is to deviations from the distribution over demonstrations because σ^2^ directly influences the variance along this distribution, as expressed by Equation (19). A small σ^2^ results in assessing positions as incorrect more often, while a high σ^2^ results in a less strict evaluation.

### 3.4. Assessing the correctness of new trajectories

The correctness of each position of a new trajectory is assessed by comparing the probability density function evaluated at that position with the probability density function evaluated at the corresponding position along the mean trajectory, which is considered by our system the best achievable trajectory, since it is the one with the highest probability under the Gaussian distribution over demonstrations.

First, the ratio

(20) $$g\left(t\right)=\frac{p\left(\tau \left(t\right)\right)}{p\left(\mu_{\tau }\left(t\right)\right)}$$

is computed for each time step *t*, where *p*(***τ***(*t*)) is the probability of position ***τ***(*t*) at time step *t* and *p*(***μ**_**τ**_*(*t*)) is the probability of position ***μ**_**τ**_*(*t*) at time step *t*. Since the highest achievable value of the Gaussian probability density function at each time step is the one achieved by the mean trajectory, *g* is a function with values between 0 and 1.

Subsequently a score

(21) $$s\left(g\left(t\right)\right)=\frac{\arctan\left(\left(g\left(t\right)+a\right)b\right)}{2c}+0.5$$

for each time step *t* is computed, where

(22) c=arctan((1+a)b).

**Figure 4:**
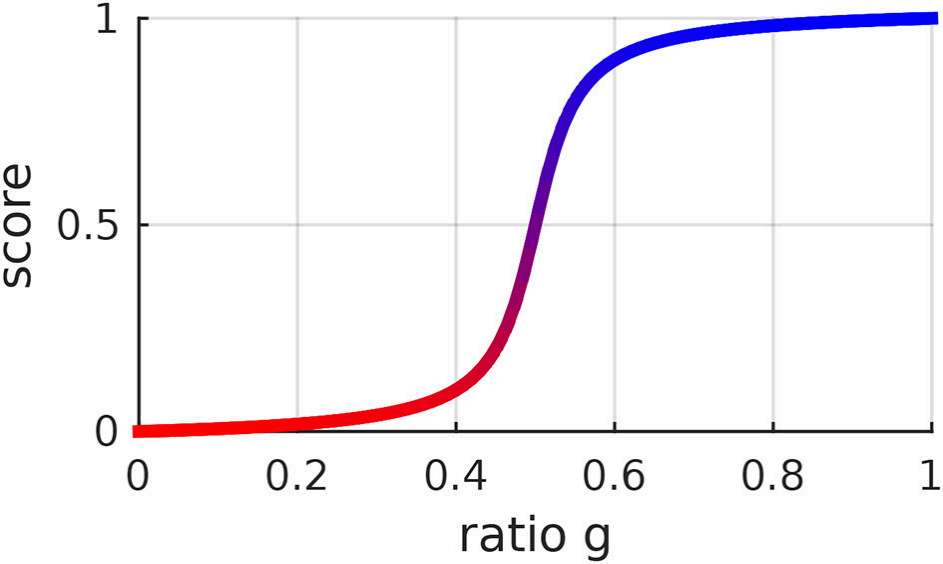
Score function of the ratio *g* between the probability of a certain position and the probability of the corresponding position along the mean trajectory. This function is determined by (21) and was designed to be 0 when *g* = 0, 1 when *g* = 1 and to monotonically increase with *g*. It is possible to change the steepness of this function by changing its hyperparameter *b*. The same color code as in this figure is used to give visual feedback to the user.

The score function *s* was designed with a few desired properties in mind. With *a* = −0.5, *s* is equal to 0 when the ratio *g* is equal to 0, it is 0.5 when *g* is 0.5 and it is 1 when *g* is 1. The score function *s* monotonically increases with *g*. Its steepness is regulated by the parameter *b*. We have been using *a* = −0.5 and *b* = 25. One could consider using other score functions, depending on the preferences of the users. The score function depicted in Figure 4 leads to a sharp distinction between right and wrong positions. One might prefer a more gradual distinction. In this work, we did not investigate what score function the users prefer nor whether certain score functions make the users learn faster. These considerations could be subject of extensive user studies.

## 4. Method to provide haptic feedback

Up to now, it has been solely discussed in this paper how to provide offline visual feedback to the user assessing the correctness of his/her movements. Here, it is presented how our framework provides online haptic feedback to the user, guiding him/her toward correct movements.

The Haption Virtuose 6D can provide force feedback to the user by simulating a virtual object attached to its end effector constituting a mass-spring-damper system. Given the mass and the inertia of the virtual object, the Virtuose API computes stiffness and damping coefficients that guarantee the stability of the system. The intensity of the force produced by this system can be rescaled by a factor denoted in this paper by ζ.

In this work, we are interested in providing feedback to the user according to a probability distribution over trajectories, which is computed as in section 3.3. If the standard deviation at a certain part of the distribution is high, the haptic device should become compliant in that region, while if the standard deviation is low, the haptic device should become stiff. The virtual object always lies along the mean trajectory of the distribution. The factor ζ can be derived from

(23) $$\frac{\zeta -\zeta_{\text{min}}}{\zeta_{\text{max}}-\zeta_{\text{min}}}=\frac{\sigma -\sigma_{\text{max}}}{\sigma_{\text{min}}-\sigma_{\text{max}}},$$

where ζ_min_ and ζ_max_ are respectively the minimum and the maximum force scaling factor. These values have been empirically defined in our experiments. The variable σ stands for the standard deviation that corresponds to the current position of the virtual object. The variables σ_min_ and σ_max_ stand for the minimum and maximum standard deviations of the distribution over trajectories. These values can be determined from a set of demonstrated trajectories. The underlying assumption behind Equation (23) is that the stiffness is the highest when the standard deviation is the minimum and the lowest when the standard deviation is the maximum. Moreover, we assume a linear dependence between ζ − ζ_min_ and σ − σ_max_. Rearranging Equation (23), we get

(24) $$\zeta =\zeta_{\text{min}}+\left(\zeta_{\text{max}}-\zeta_{\text{min}}\right)\left(\frac{\sigma -\sigma_{\text{max}}}{\sigma_{\text{min}}-\sigma_{\text{max}}}\right).$$

The closest point along the mean trajectory that is not further away from the previous position of the virtual object than a certain threshold becomes the new position of the virtual object. This threshold is especially necessary when dealing with convoluted trajectories to avoid large sudden variations in the position of the virtual object.

In situations where there are no expert demonstrations available, but there is a performance measurement of the trajectories, it is possible to use reinforcement learning to improve the distribution over trajectories. Such a situation could be found in a teleoperation scenario, where an optimization problem with multiple objectives may have to be solved, accounting for distances to via points, distances to obstacles and other performance measurements. In the next section, a novel reinforcement learning algorithm is presented to address such problems.

## 5. Relevance weighted policy optimization

We are interested in enabling a haptic device to assist a human in a task also when the available demonstrations are suboptimal. As it will be presented in section 6.2, our particular task is to move an object in a virtual environment from a start position to an end position through a window in a wall. We have defined three objectives to determine the performance of solutions to this task: distance to the start position, distance to the center of the window and distance to the end position. An optimal policy for this task is a trajectory that begins at the start position, passes through the center of the window and reaches the end position. This problem can be decomposed into three subproblems w.r.t. which a policy parameter can be more or less relevant. Therefore, in this section, a new policy search method is explained, which identifies the relevance of each policy parameter to each subproblem in order to improve the learning of the global task. This method makes use of Reward-weighted Regression (Peters and Schaal, 2007). The basic idea of this method is to first find out how much each policy parameter influences each objective. Subsequently, this information is used to optimize the policy with respect to the objectives. In our particular application, the policy parameters are the elements of the weight vector ***w*** as in Equation (14).

### 5.1. Learning relevance functions

Our approach to answering how much each policy parameter influences each objective consists of learning a relevance function *f*_*o*_ for each objective *o*. The argument of this function is an index identifying a policy parameter. In other words, a relevance function *f*_*o*_(*n*) evaluated for policy parameter indexed by *n* represents how relevant this parameter is to the objective indexed by *o*. In order to learn this function, in this paper, it is assumed that a relevance function can be represented by a weighted sum of basis functions with lower bound 0 and upper bound 1 as follows:

(25) $$f_{o}\left(n\right)=\{\begin{array}{ll} 0, & \text{if }\rho^{T}\phi \left(n\right)\leq 0\\ 1, & \text{if }\rho^{T}\phi \left(n\right)\geq 1\\ \rho^{T}\phi \left(n\right), & \text{otherwise}, \end{array},$$

where ***ρ*** is a vector of weights *ρ*_*i*_ for the basis functions *ϕ_i_* and $\phi \left(n\right)={\left[\phi_{1}\left(n\right),\phi_{2}\left(n\right),\cdots \;,\phi_{I}\left(n\right)\right]}^{T}$. It will become clear in the remainder of this section why the lower bound of a relevance function is 0 and its upper bound is 1.

The basis functions are

(26) $$\phi_{i}\left(n\right)=\frac{1}{\exp\left(-k\left(n-m_{i}\right)\right)},$$

(27) $$\phi_{I}=1,$$

with *i* ∈ {1, 2, ⋯ , *I*}, where *I* is the total number of basis functions for the relevance function, *n* is an index representing one of the policy parameters, *k* is a scalar determining steepness and *m*_*i*_ is a scalar determining the midpoint of the logistic basis function with index *i*.

**Figure 5:**
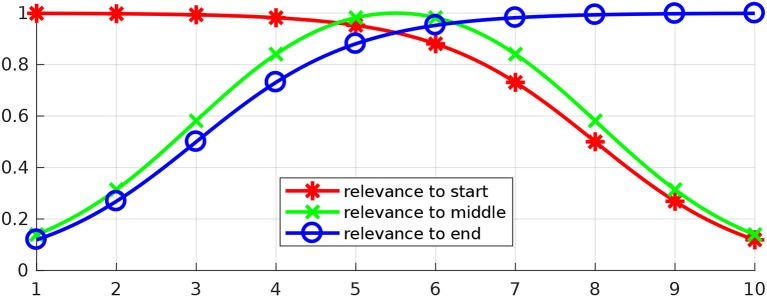
Three examples of relevance functions. Let us assume that our goal is to optimize a certain movement with respect to an objective at the beginning of the movement, an objective in the middle and an objective in the end. Let us further assume that the movement to be optimized can be determined by 10 parameters and that the first parameters (close to 1) have higher influence over the beginning of the movement, while the last ones (close to 10) have higher influence over the end. The image depicts potentially suitable relevance functions for each of the objectives in this problem.

These basis functions have been chosen because weighted combinations of them lead to reasonable relevance functions. For example, three relevance functions that can be constructed with the proposed basis functions are depicted in Figure 5. The depicted relevance functions determine how each of the parameters determining a movement influences objectives at the beginning of the movement, in the middle or in the end. These relevance functions are

(28) $$f_{\text{start}}\left(n\right)=\phi_{3}\left(n\right)-\phi_{2}\left(n\right),$$

(29) $$\begin{array}{l} f_{\text{middle}}\left(n\right)=\frac{1}{\underset{n}{\max}\;(\phi_{1}\;(n)\;-\;\phi_{2}\;\left(n\right))}\phi_{1}\left(n\right)\\ \;-\frac{1}{\underset{n}{\max}\;(\phi_{1}\;\left(n\right)\;-\;\phi_{2}\;\left(n\right))}\phi_{2}\left(n\right), \end{array}$$

(30) $$f_{\text{end}}\left(n\right)=\phi_{1}\left(n\right),$$

where the basis functions are

(31) $$\phi_{1}\left(n\right)=\frac{1}{\exp\left(-\left(n-3\right)\right)},$$

(32) $$\phi_{2}\left(n\right)=\frac{1}{\exp\left(-\left(n-8\right)\right)},$$

(33) $$\phi_{3}\left(n\right)=1.$$

In this framework, learning a relevance function with respect to a certain objective means finding a vector ***ρ*** that leads to a high variability in the value of that objective and to a low variability in the values of other objectives. How a relevance function influences the variability in the values of an objective will be made explicit in the following.

First, a Gaussian distribution $N\left(\mu_{\rho },\Sigma_{\rho }\right)$ over ***ρ*** is initialized with a certain mean ***μ_ρ_*** and a certain covariance matrix **Σ**_***ρ***_. Subsequently, parameter vectors ***ρ*** are sampled from this distribution and, for each sample, the relevance function *f*_*o*_ is computed using Equation (25).

Let us now assume that there is an initial Gaussian probability distribution $N\left(\mu_{w},\Sigma_{w}\right)$ over the policy parameters ***w***. The mean ***μ_*w*_*** and the covariance matrix **Σ_*w*_** can be computed from an initial set of demonstrations or determined by the user.

For each *f*_*o*_ computed with the sampled vectors ***ρ***, our algorithm generates samples of the policy parameters ***w*** from the distribution $N\left(\mu_{w},\Sigma_{w}{\;}^{f_{o}}\right)$, where

(34) $$\begin{array}{l} \Sigma_{w}{\;}^{f_{o}}=\left[\begin{array}{cccc} \sigma_{w_{1}}^{2}f_{o}\left(1\right) & 0 & \cdots & 0\\ 0 & \sigma_{w_{2}}^{2}f_{o}\left(2\right) & \cdots & 0\\ \vdots & \vdots & \ddots & \vdots \\ 0 & 0 & \cdots & \sigma_{w_{N}}^{2}f_{o}\left(N\right) \end{array}\right] \end{array}$$

and $\sigma_{w_{n}}^{2}$, ∀*n* ∈ {1, 2, ⋯ , *N*}, are the variances in the diagonal of the matrix **Σ_*w*_**. In other words, the policy parameters are sampled in such a way that their original variance is weighted with a relevance coefficient. The higher the relevance of a parameter, the larger the range of values for that parameter among the samples.

Each sampled vector of policy parameters ***w*** determines a policy with a corresponding value for each objective. In our teleoperation scenario, for example, each policy parameter vector ***w*** determines a trajectory, which has a certain distance to the start position, a certain distance to the center of the window and a certain distance to the end position. Given these objective values, our algorithm computes a reward function

(35) $$R_{\rho ,o}=\exp\left(\beta_{\text{relevance}}\left(\sigma_{o}-\sum_{i\neq o}\sigma_{i}\right)\right),$$

where σ_*o*_ is the standard deviation of the values for objective *o* and σ_*i*_ with *i* ≠ *o* is the standard deviation of the values for the other objectives. The scalar *β*_relevance_ can be determined with line search.

Parameters ***ρ*** determining suitable relevance functions *f*_*o*_ result in higher reward *R*_***ρ***,*o*_ because the range of values for the parameters that mainly affect objective *o* will be high, producing a high standard deviation of the values for that objective. Moreover, the range of values for the parameters that mainly affect the other objectives will be low, producing a low standard deviation of the values for the other objectives.

Finally, Reward-weighted Regression (RWR) is used to learn the relevance parameters ***ρ***. RWR is an iterative algorithm that finds the best Gaussian distribution over parameters of interest (in the particular case of optimizing the relevance functions, the parameters of interest are given by ***ρ***) to maximize the expected reward, given samples from the Gaussian distribution of the previous iteration. In order to do so, RWR solves the optimization problem

(36) $$\text{\{}\mu_{\rho }^{k+1},\;\Sigma_{\rho }^{k+1}\text{\}}=\underset{\text{\{}\mu_{\rho },\Sigma_{\rho }\text{\}}}{\text{arg max}}\sum_{i=1}^{S}R_{\rho ,o,i}N\left(\rho_{i};\mu_{\rho },\;\Sigma_{\rho }\right)$$

at each iteration, where *S* is the number of sampled parameter vectors ***ρ***_*i*_ from the previous distribution $N\left(\mu_{\rho }^{k},\Sigma_{\rho }^{k}\right)$. The solution to this optimization problem is

(37) $$\mu_{\rho }^{k+1}=\frac{\sum_{i=1}^{S}R_{\rho ,o,i}\rho_{i}}{\sum_{i=1}^{S}R_{i}},$$

(38) $$\Sigma_{\rho }^{k+1}=\frac{\sum_{i=1}^{S}R_{\rho ,o,i}\left(\rho_{i}-\mu_{\rho }^{k}\right){\left(\rho_{i}-\mu_{\rho }^{k}\right)}^{T}}{\sum_{i=1}^{S}R_{\rho ,o,i}}.$$

This procedure is repeated until convergence of *R*_***ρ***,*o*_ has been reached to learn a relevance function *f*_*o*_ for each objective *o*. The parameters determining the relevance functions *f*_*o*_ are given by the vector ***μ**_**ρ**_* computed in the last iteration. After this iterative procedure is finished, our algorithm computes $f_{o}\left(n\right)/\underset{n}{\max}f_{o}\left(n\right)$, ∀*n* ∈ {1, 2, ⋯ , *N*}, and assigns this value to *f*_*o*_(*n*). This last step makes the maximum value of *f*_*o*_ be not less than 1 and helps the exploration in the policy optimization phase, which will be discussed in the next section. Algorithm 4 presents an informal description of the relevance learning algorithm.

**Algorithm 4:**
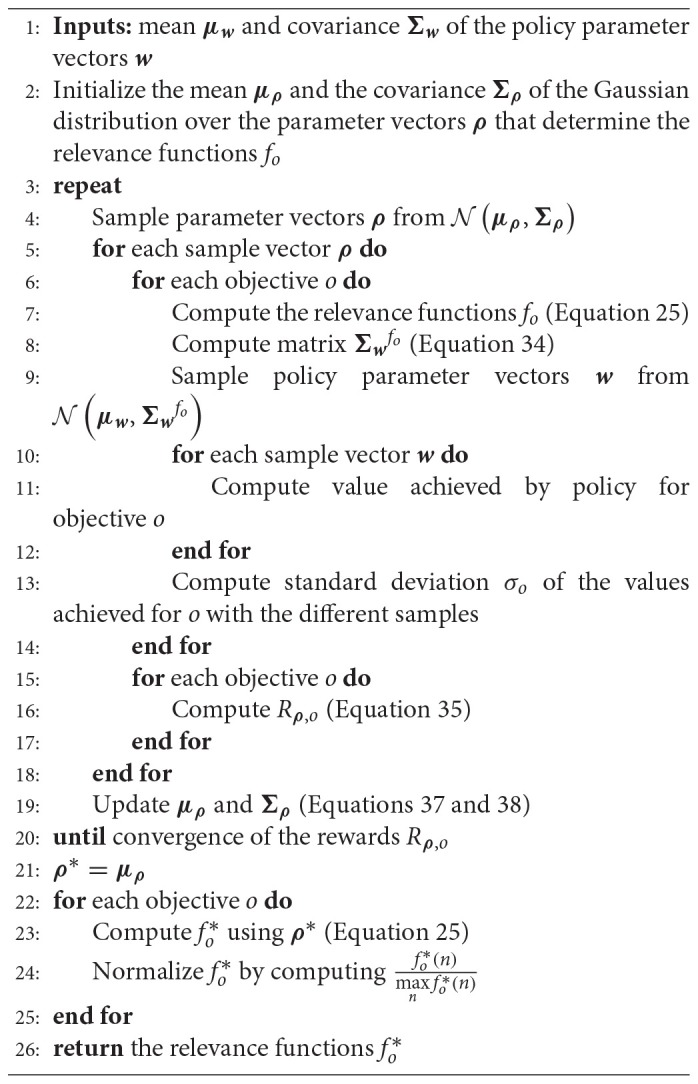
Learning Relevance Functions

### 5.2. Policy optimization using relevance functions

Now that a relevance function for each objective has been learned, our algorithm uses this information to optimize a policy with respect to each objective. As in section 5.1, it is assumed here that there is an initial Gaussian probability distribution $N\left(\mu_{w},\Sigma_{w}\right)$ over the policy parameters ***w***.

For each objective *o*, our algorithm samples policy parameters ***w*** from the distribution $N\left(\mu_{w},\;\Sigma_{w}{\;}^{f_{o}^{*}}\right)$, where

(39) $$\begin{array}{l} \Sigma_{w}{\;}^{f_{o}^{*}}=\left[\begin{array}{cccc} \sigma_{w_{1}}^{2}f_{o}^{*}\left(1\right) & 0 & \cdots & 0\\ 0 & \sigma_{w_{2}}^{2}f_{o}^{*}\left(2\right) & \cdots & 0\\ \vdots & \vdots & \ddots & \vdots \\ 0 & 0 & \cdots & \sigma_{w_{N}}^{2}f_{o}^{*}\left(N\right) \end{array}\right] \end{array}$$

and $f_{o}^{*}$ is the learned relevance function with respect to the objective *o*. Therefore, the policy parameters ***w*** are sampled from a Gaussian distribution where the original variances $\sigma_{w_{n}}^{2}$ are weighted with the learned relevance function. This procedure means that a larger range of values will be sampled for the policy parameters *w*_*n*_ that are more relevant to the objective *o* and a smaller range of values will be sampled for the policy parameters *w*_*n*_ that are less relevant to this objective.

For each sampled vector of policy parameters ***w***_*i*_, the reward *R_**w**,o,i_* associated with the objective *o* is computed. These objectives and rewards depend on the problem. An objective might be for instance to achieve a certain goal position, in which case the reward could be a non-negative function monotonically decreasing with the distance to the goal position. In our particular teleoperation problem, the reward associated with the objective of being close to the start position is given by *R* = exp(−*β*_policy_*d*_start_), where *d*_start_ is the distance between the first position of the trajectory and the position where the trajectories should start.

Our algorithm uses once again RWR. This time, RWR is used to maximize the expected reward with respect to ***μ**_**w**_* and $\Sigma_{w}{\;}^{f_{o}^{*}}$. This maximization is done iteratively according to

(40) $$\text{\{}\mu_{w}^{k+1},C^{k+1}\text{\}}=\underset{\text{\{}\mu_{w},\Sigma_{w}{\;}^{f_{o}^{*}}\text{\}}}{\text{arg max}}\sum_{i=1}^{S}R_{w,o,i}N\left(w_{i};\;\mu_{w},\;\Sigma_{w}{\;}^{f_{o}^{*}}\right),$$

where *S* is the number of sampled policy parameter vectors ***w***_*i*_ from the previous distribution $N\left(w;\mu_{w}^{k},\Sigma_{w}{\;}^{f_{o}^{*}k}\right)$.

The solution to Equation (40) is given by

(41) $$\mu_{w}^{k+1}=\frac{\sum_{i=1}^{S}R_{w,o,i}w_{i}}{\sum_{i=1}^{S}R_{w,o,i}},$$

(42) $$C^{k+1}=\frac{\sum_{i=1}^{S}R_{w,o,i}\left(w_{i}-\mu_{w}^{k}\right){\left(w_{i}-\mu_{w}^{k}\right)}^{T}}{\sum_{i=1}^{S}R_{w,o,i}}.$$

In each iteration, after applying Equations (41) and (42), our algorithm updates the variances of each policy parameter $\sigma_{w_{n}}^{2}$ with

(43) $$\sigma_{w_{n},k+1}^{2}=(1-f_{o}\left(n\right))\;\sigma_{w_{n},k}^{2}+f_{o}\;(n)\;C_{nn}^{k+1},$$

where $\sigma_{w_{n},k}^{2}$ is the previous variance of policy parameter *w*_*n*_ and ${\text{C}}_{nn}^{k+1}$ is the *n*th element along the main diagonal of the matrix **C**^*k*+1^. This equation has the effect of keeping the previous variance of the parameters that are less relevant to the objective *o* while updating the variance of the parameters that are more relevant to this objective. The algorithm then uses $\sigma_{w_{n},k+1}^{2}$ to compute $\Sigma_{w}{\;}^{f_{o}^{*}k+1}$ as in Equation (39).

Finally, Equation (43) justifies the lower bound of 0 and the upper bound of 1 for the relevance function. The closer the relevance of policy parameter *w*_*n*_ is to 0, the closer the updated variance of this parameter is to the previous variance $\sigma_{w_{n},k}^{2}$. The closer the relevance of policy parameter *w*_*n*_ is to 1, the closer the updated variance of this parameter is to ${\text{C}}_{nn}^{k+1}$. In other words, the previous variance of irrelevant policy parameters is preserved, while the variance of relevant policy parameters is updated. Algorithm 5 presents an informal description of the algorithm for policy optimization using relevance functions.

**Algorithm 5:**
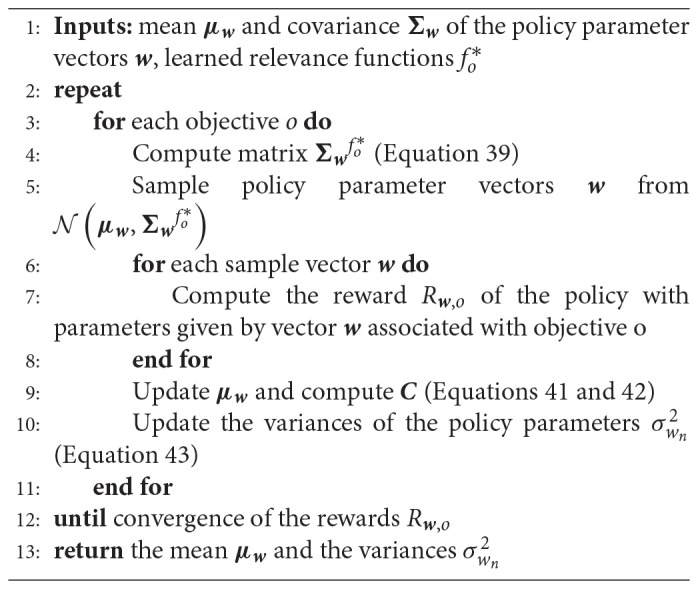
Policy Optimization using Relevance Functions

### 5.3. Example of policy optimization with relevance weighting

**Figure 6:**
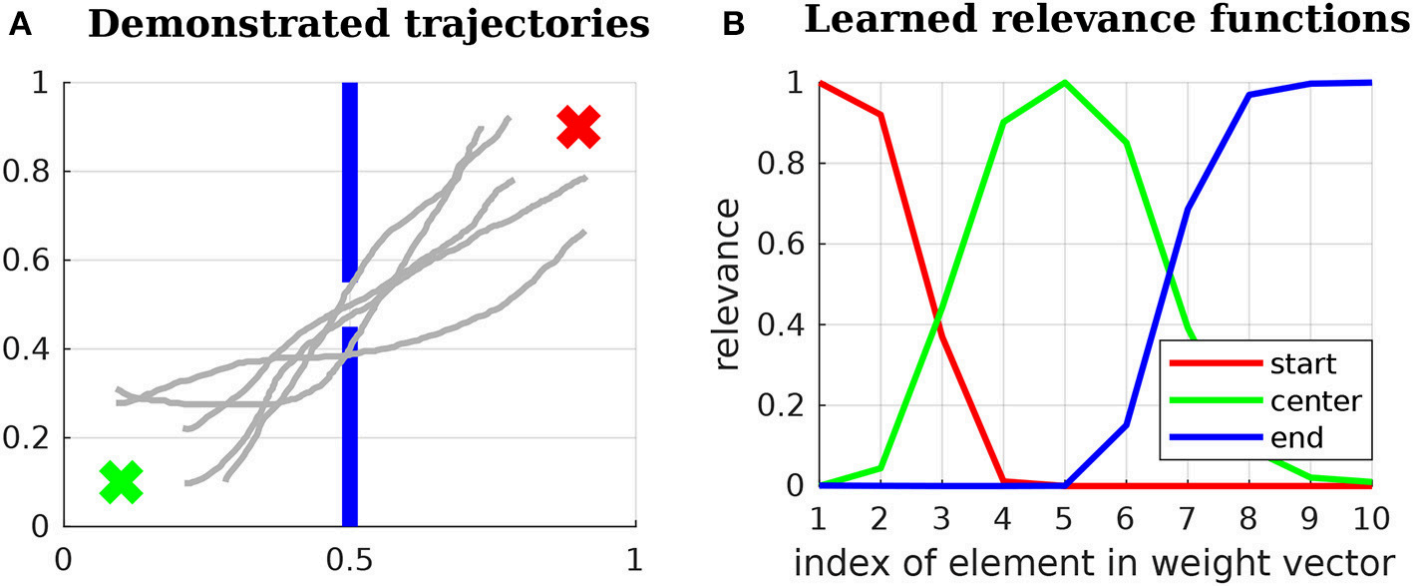
**(A)** 2D problem used to explain the proposed Relevance Weighted Policy Optimization (RWPO) algorithm. The green x at the lower left corner of the image represents the start position. The blue lines in the middle represent a wall with a window in the center. The red x at the upper-right corner represents the end position. The goal of our algorithm is, given a few initial trajectories (depicted in light gray), to find a distribution over trajectories that begin at the start position, pass through the center of the window and reach the end position. **(B)** Learned relevance functions for the 2D problem. The learned relevance functions show that policy parameters close to *w*_1_ are more important for beginning at the start position, policy parameters around *w*_5_ are more important to pass through the center of the window and policy parameters close to *w*_10_ are more important to reach the end position.

In order to make the proposed Relevance Weighted Policy Optimization algorithm more clear, we present an example using the 2D scenario depicted in Figure 6A. This scenario is composed of a start position, a wall with a window and an end position. Given the initial trajectories depicted in Figure 6A, the goal of our algorithm is to find a distribution over trajectories that begin at the start position, pass through the center of the window and reach the end position.

**Figure 7:**
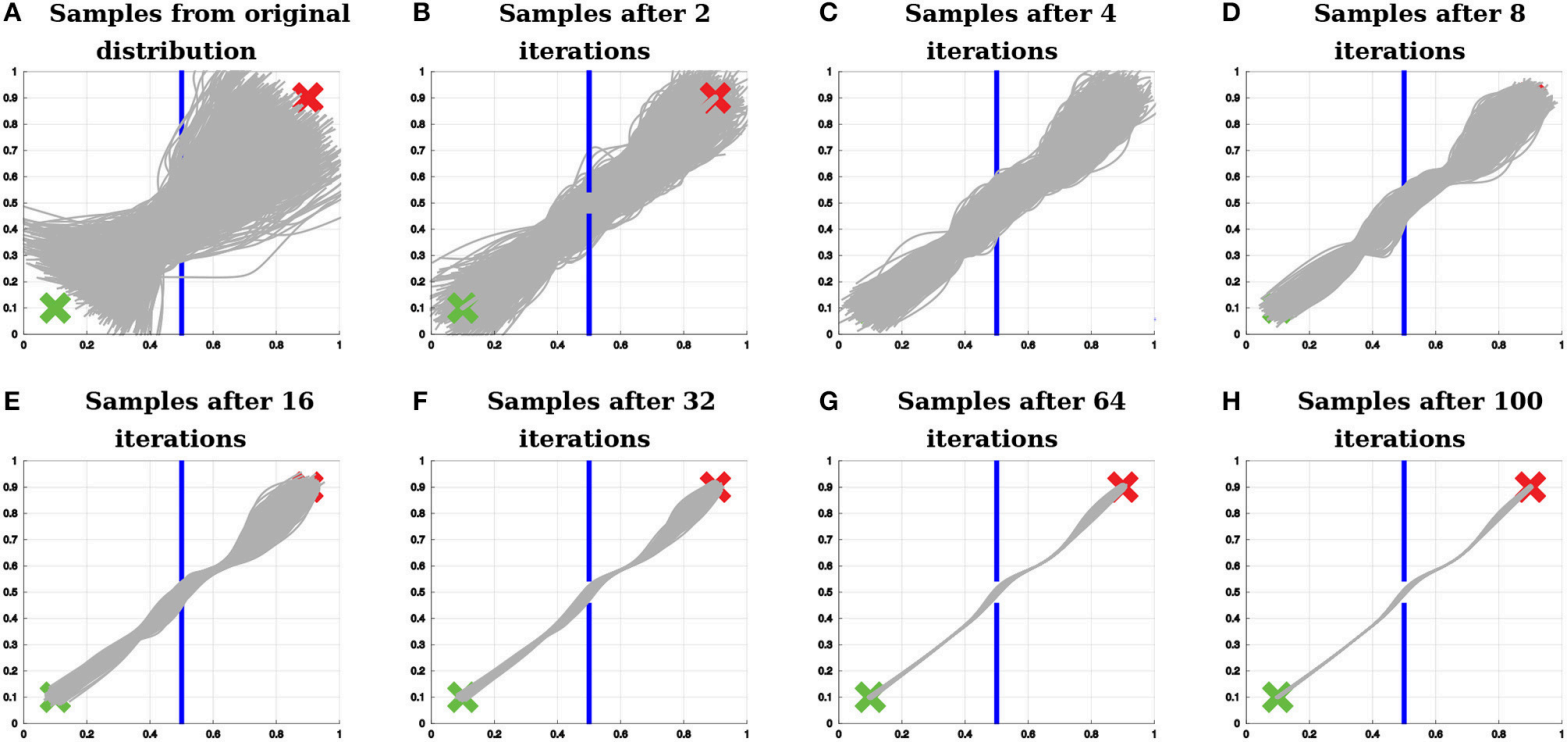
Example of policy search with relevance weighting. The proposed algorithm finds a distribution over trajectories that start and end at the correct positions (represented by the green x and by the red x, respectively) and do not hit the wall (represented by the blue lines).

**Figure 8:**
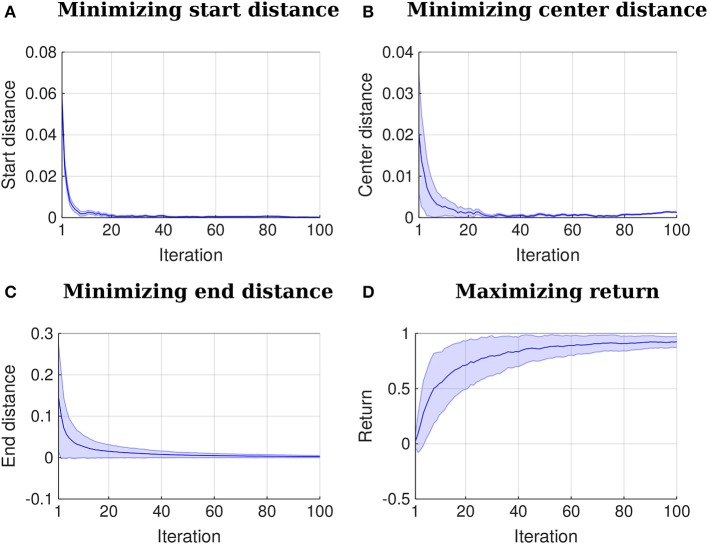
Iteration vs. distances and iteration vs. return. The plots represent mean and two times the standard deviation. All the distances to the points of interest decrease to 0 or close to it with the number of iterations. A return function given by exp(−*β*_policy_(*d*_start_ + *d*_center_ + *d*_end_)) increases with the number of iterations. Here, *β*_policy_ is a parameter which can be determined with line search, *d*_start_ is the distance to the start, *d*_center_ is the distance to the center and *d*_end_ is the distance to the end.

First, the algorithm aligns the initial trajectories in time and computes the parameters ***w*** for each of them using Equation (16). Subsequently, the relevance functions for start position, center and end position are learned as in section 5.1. An example of learned relevance functions is depicted in Figure 6B. After learning the relevance functions, the algorithm uses the procedure explained in section 5.2 to learn a policy that satisfies the three above-stated objectives. Figure 7 shows how the distribution over trajectories changes with the number of iterations of the algorithm. The distances to start, center and end positions decrease with the number of iterations and the return exp(−*β*_policy_(*d*_start_ + *d*_center_ + *d*_end_)) increases, as depicted by Figure 8. Here, *β*_policy_ is a parameter which can be determined with line search, *d*_start_ is the distance to the start, *d*_center_ is the distance to the center and *d*_end_ is the distance to the end.

Relevance Weighted Policy Optimization implements policy search for each objective sequentially. For each objective, the algorithm samples a larger range of values for the parameters that are more relevant to that objective, while sampling values close to the mean for the parameters that are less relevant. Subsequently, the algorithm optimizes the mean and the variances of the policy parameters given the samples. After optimization, the mean and the variance of the parameters that matter more to that objective are updated, while the mean and the variance of parameters that matter less remain similar to the previous distribution. The algorithm does not require defining a reward function with different weights for the different objectives, which can be time-consuming and ineffective. Moreover, at each iteration, when optimizing the distribution over policy parameters with respect to a certain objective, the algorithm does not accidentally find solutions that are good according to this objective, but bad according to the other objectives because only the mean and the variance of the parameters that matter change substantially. The mean and the variance of the other parameters remain close to the mean and the variance of the previous distribution.

**Figure 9:**
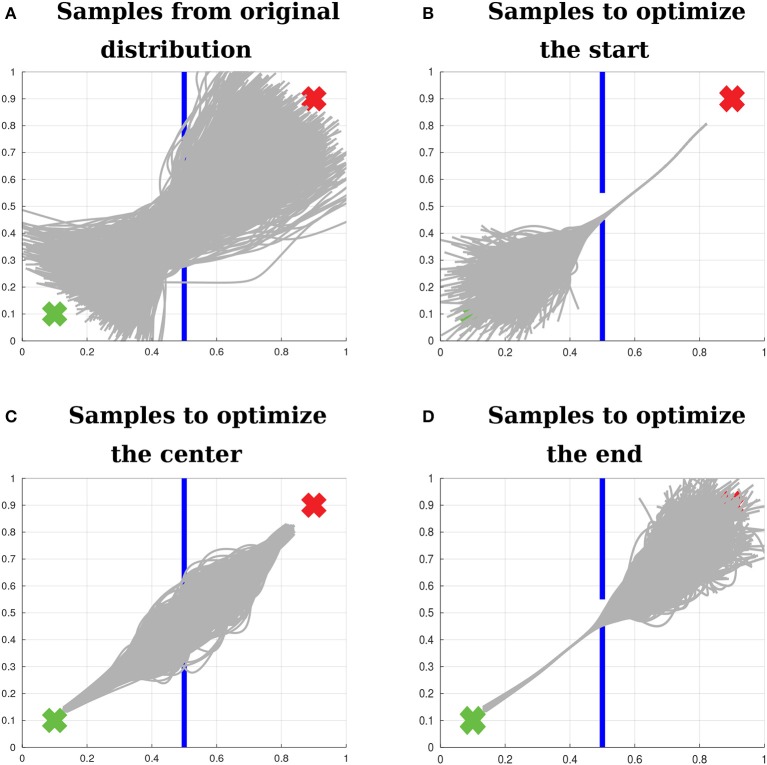
Sampling with relevance weighting. **(A)** Samples from the original distribution. **(B)** Samples to optimize the distribution over trajectories with respect to beginning at the start position. **(C)** Samples to optimize the distribution over trajectories with respect to passing through the center of the window. **(D)** Samples to optimize the distribution over trajectories with respect to reaching the end position. The proposed algorithm explores for each objective a large range of values for the policy parameters that are relevant to that objective, while sampling values close to the mean for the other policy parameters. The variance of the irrelevant parameters is recovered according to Equation (43). Therefore, after optimizing for each objective, the distribution over the relevant parameters is updated, while the distribution over the irrelevant parameters is preserved.

Figure 9 exemplifies how the algorithm samples trajectories in the 2D teleoperation problem. Figure 9A shows samples from the original distribution. Figure 9B shows samples of the first iteration of the algorithm right before optimizing for beginning at the start position. Figure 9C depicts the next step, still in the first iteration, after the first optimization for starting at the start position and before optimizing for passing through the center of the window. Finally, Figure 9D shows samples at the first iteration of the algorithm, right before optimizing for reaching the end position.

**Figure 10:**
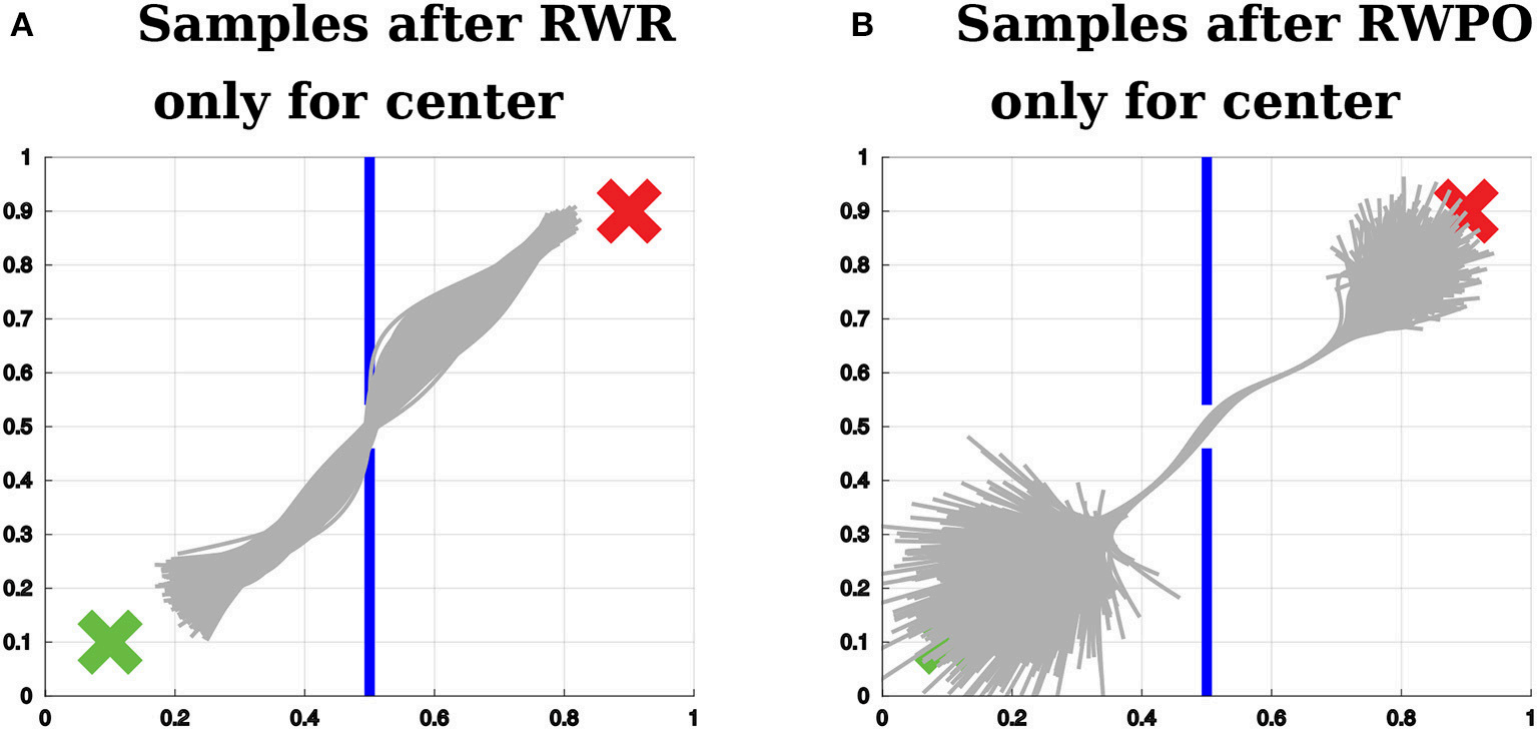
**(A)** Sample trajectories after using Reward-weighted Regression (RWR) to optimize the distribution over trajectories with respect to passing through the center of the window. **(B)** Sample trajectories after using Relevance Weighted Policy Optimization (RWPO) to optimize the distribution over trajectories with respect to the same objective, using the same reward function. In contrast to RWR, RWPO finds a better policy to avoid hitting the wall and does not shrink the variance of parts of the trajectories that are far away from the region of interest.

Figure 10A shows a distribution over trajectories learned by Reward-weighted Regression (RWR) optimizing only for passing through the center of the window. Figure 10B shows the solution of Relevance Weighted Policy Optimization (RWPO) for this same optimization problem. RWPO's solution achieves the objective with higher accuracy and preserves a large variance for parts of the trajectory that do not influence the objective.

**Figure 11:**
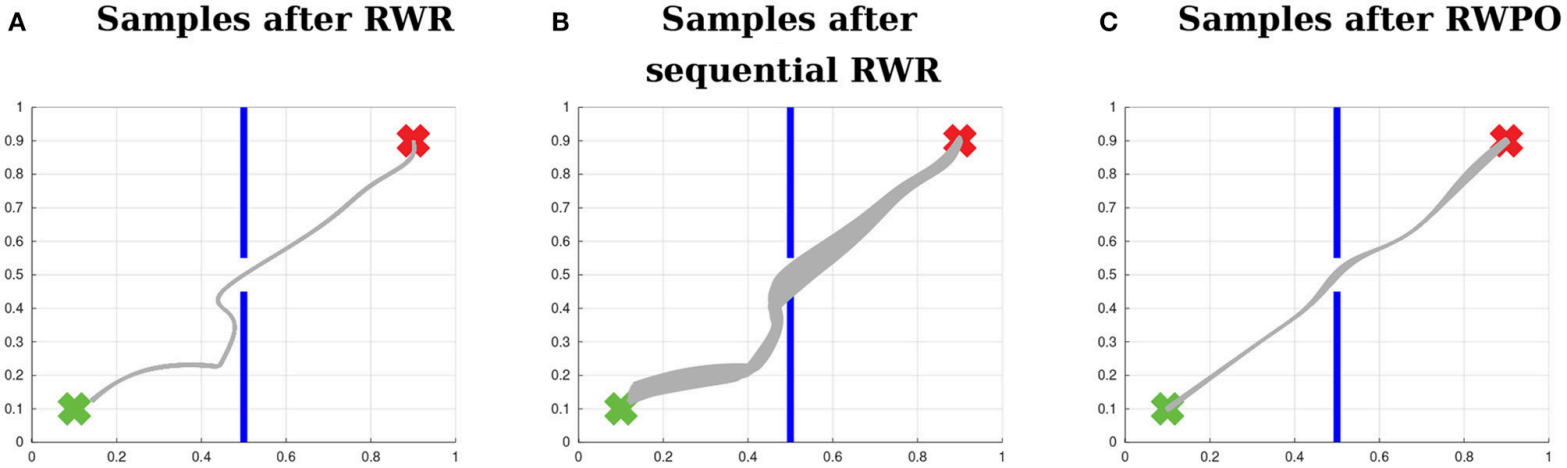
Comparison between Reward-weighted Regression (RWR), sequential Reward-weighted Regression (sRWR) and Relevance Weighted Policy Optimization (RWPO). This time, the three algorithms optimize the distribution over trajectories with respect to all objectives. **(A)** Distribution after optimization with RWR, which uses a single reward function with a term for each objective. **(B)** Distribution after optimization using sRWR, which optimizes for each objective sequentially and has a reward function for each objective. **(C)** Distribution after optimization using RWPO, which uses the concept of relevance functions and optimizes for each objective sequentially with a reward function for each objective.

Finally, Figure 11 shows a comparison between Reward-weighted Regression (RWR), sequential Reward-weighted Regression (sRWR) and Relevance Weighted Policy Optimization (RWPO). RWR used here a reward function of the form *R* = exp(−*β*_policy_(*d*_start_ + *d*_center_ + *d*_end_)), while sRWR and RWPO used one reward function for each objective:

(44) $$R_{\text{start}}=\exp\left(-\beta_{\text{policy}}\left(d_{\text{start}}\right)\right),$$

(45) $$R_{\text{center}}=\exp\left(-\beta_{\text{policy}}\left(d_{\text{center}}\right)\right),$$

(46) $$R_{\text{end}}=\exp\left(-\beta_{\text{policy}}\left(d_{\text{end}}\right)\right).$$

## 6. Experiments

We demonstrate our method to assist the practice of motor skills by humans with the task of writing Japanese characters. Moreover, an experiment involving a haptic device, the Haption Virtuose 6D, demonstrates how our method can be used to give haptic feedback to the user, guiding him/her toward correct movements according to certain performance criteria even in the absence of expert demonstrations.

### 6.1. Teaching japanese characters

In these experiments, first, a human provided with a computer mouse 10 demonstrations of a certain Japanese character composed of multiple strokes. Our system aligned these demonstrations in space and time. Afterward, a human provided a new trajectory. This new trajectory was also aligned in space and time by our system with respect to the demonstrations. Once all the demonstrations and the new trajectory had been time-aligned, our system computed a probability distribution over the demonstrations. Based on the probability density function evaluated at each position of the new trajectory in comparison to the probability density function evaluated at corresponding positions along the mean trajectory, our system computed a score. This score was then used to highlight parts of the new trajectory that do not fit the distribution over demonstrations with a high probability.

**Figure 12:**
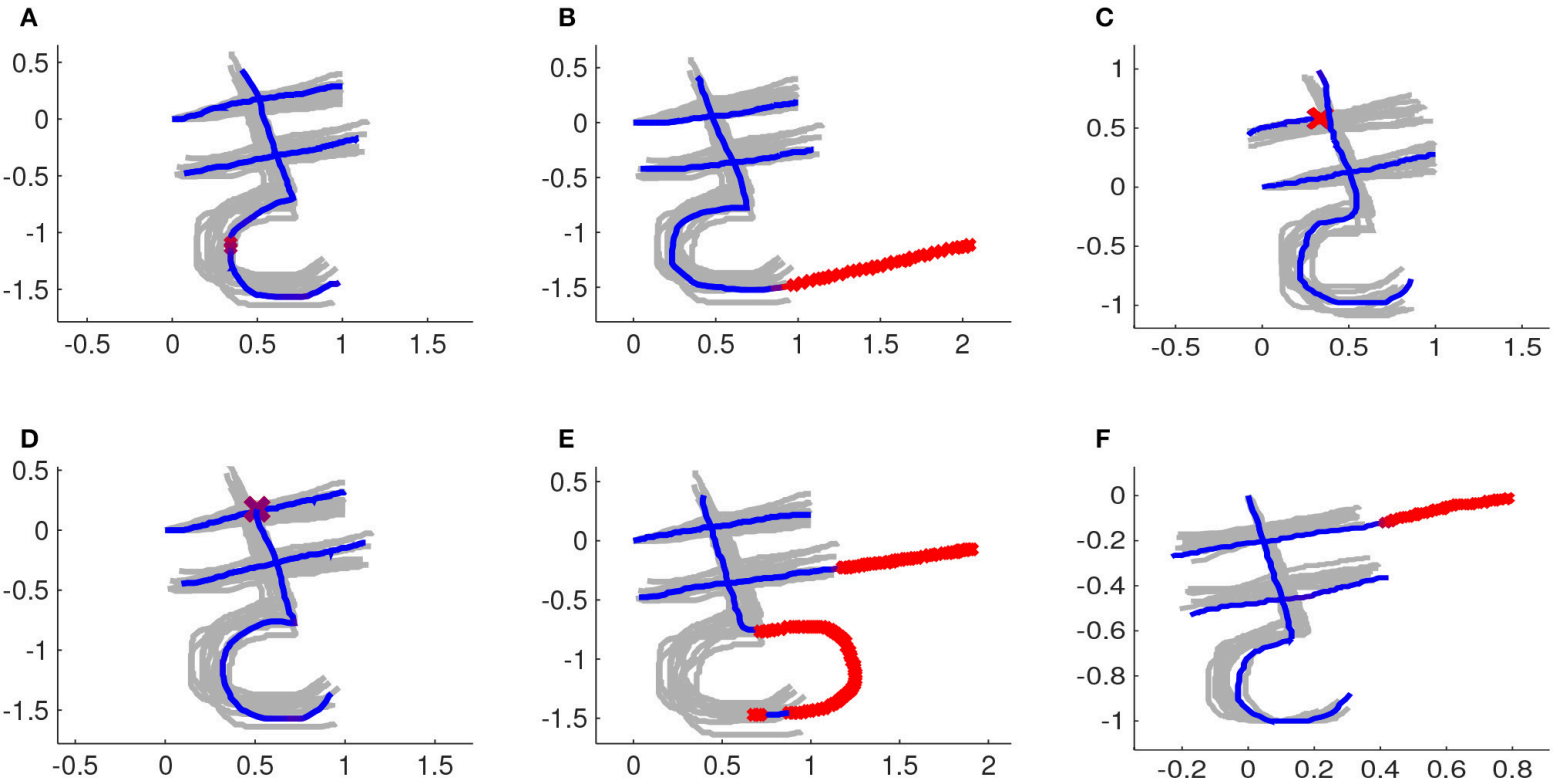
The demonstrations after rescaling, repositioning and time-alignment are depicted in light gray. Parts of a new trajectory that are considered correct are depicted in blue. Parts of a new trajectory that are considered wrong are marked with red x's . **(A)** Instance with a small mistake in the third stroke. **(B)** Third stroke goes further than it should. **(C)** First stroke is too short. **(D)** Third stroke starts too low. **(E)** Second stroke is too long and third stroke has its arch in the wrong direction. **(F)** First stroke is too long.

Figure 12 shows some examples of feedbacks provided by our system. The new trajectory provided by the user is also aligned in space and time. Therefore the absolute position of his/her character and its scale are not relevant. The speed profile of the new trajectory can also be different from the speed profile of the demonstrations. Figure 12 shows the new trajectories already after alignment in space and time.

### 6.2. Haptic feedback

When learning complex movements in a 3D environment or perhaps when manipulating objects, haptic feedback may give the human information about how to adapt his/her movements that would be difficult to extract only from visual feedback. Therefore, we investigated how to give haptic feedback based on a probability distribution over trajectories possibly provided by an instructor or resulting from a reinforcement learning algorithm. This study was carried out in accordance with the recommendations of the Declaration of Helsinki in its latest version. The protocol was approved by the Ethical Committee of the Technische Universität Darmstadt. All participants provided written informed consent before participation.

In this user experiment, users had to use the Haption Virtuose 6D device to teleoperate a small cube in a 3D environment (See Figure 14A). The users were instructed to begin at the position marked by the yellow sphere, pass through the center of the window in the wall and end at the position marked by the blue sphere. Moreover, it was allowed, at any time, to rotate the virtual environment, zoom in and zoom out using the computer mouse. Five users took part in our experiments: two females and three males, between 27 and 29 years old. Three users had not used the Virtuose 6D before, while two users did have some experience with it.

**Figure 13:**
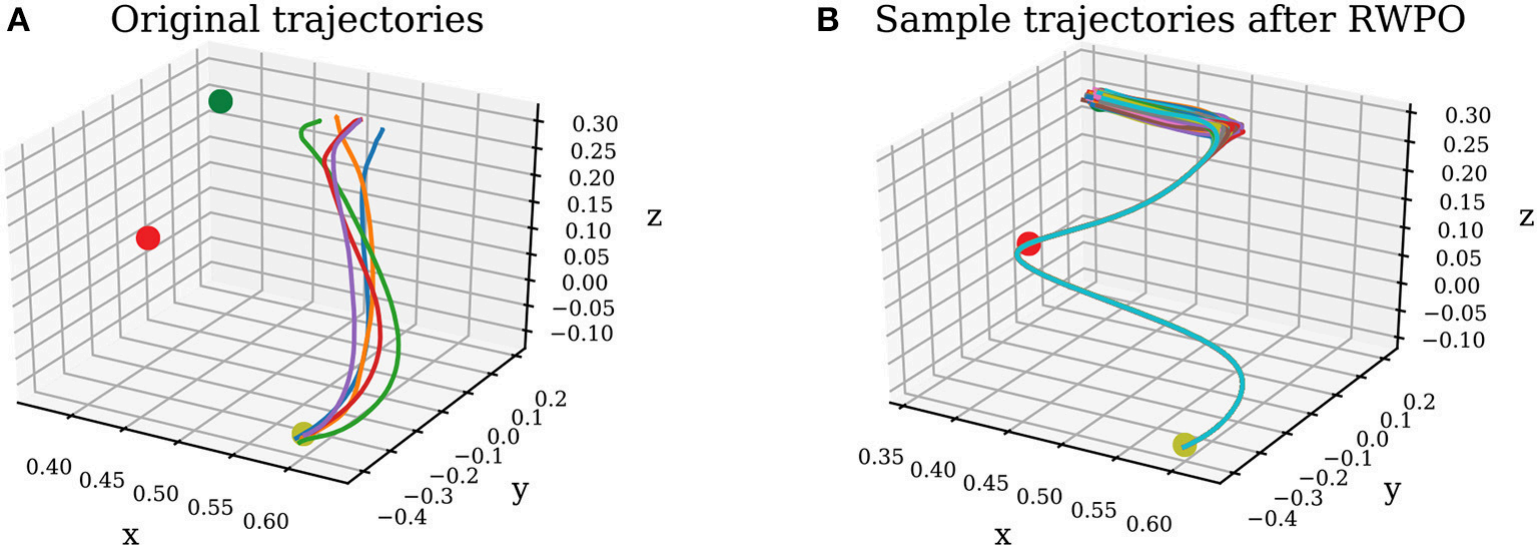
**(A)** Original trajectories. **(B)** Sample trajectories after Relevance Weighted Policy Optimization (RWPO).

**Figure 14:**
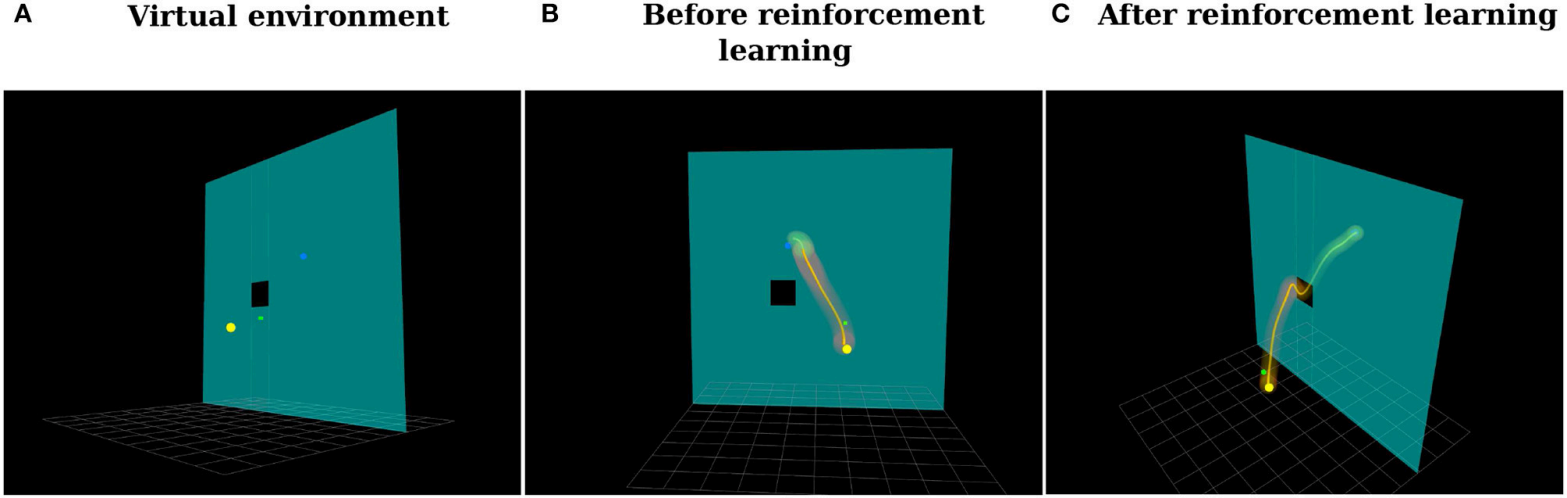
**(A)** Virtual environment. The goal of the user is to teleoperate the green cube to move it from the position marked by the yellow sphere to the position marked by the blue sphere through the window to not hit the wall. **(B)** Distribution over trajectories before reinforcement learning. **(C)** Distribution over trajectories after reinforcement learning using the proposed Relevance Weighted Policy Optimization (RWPO) algorithm.

In the first phase of the experiment, users tried to perform the task ten times without force feedback. Right before each trial, the user pressed a button on the handle of the haptic device, indicating to our system when to start recording the trajectory. By pressing this same button another time, by the end of the trajectory, the user indicated to our system when to finish recording. The users were then instructed to move the cube back to the start position and perform another trial. This procedure would be repeated until ten trajectories had been recorded. Afterward, our system would align them in time and compute a probability distribution over them. Figure 14B shows a visualization of the distribution over trajectories of one user after this phase. Subsequently, our system optimized this distribution over trajectories using the proposed Relevance Weighted Policy Optimization (RWPO) algorithm. An example of trajectories before and after RWPO is depicted in Figure 13. Figure 14C shows the optimized distribution over trajectories given the initial distribution shown in Figure 14B. After optimizing the distribution over trajectories, our system used it to give force feedback to the user according to the method explained in section 4. The users were requested to try to perform the task with force feedback ten times using the aforementioned procedure to record the trajectories.

**Figure 15:**
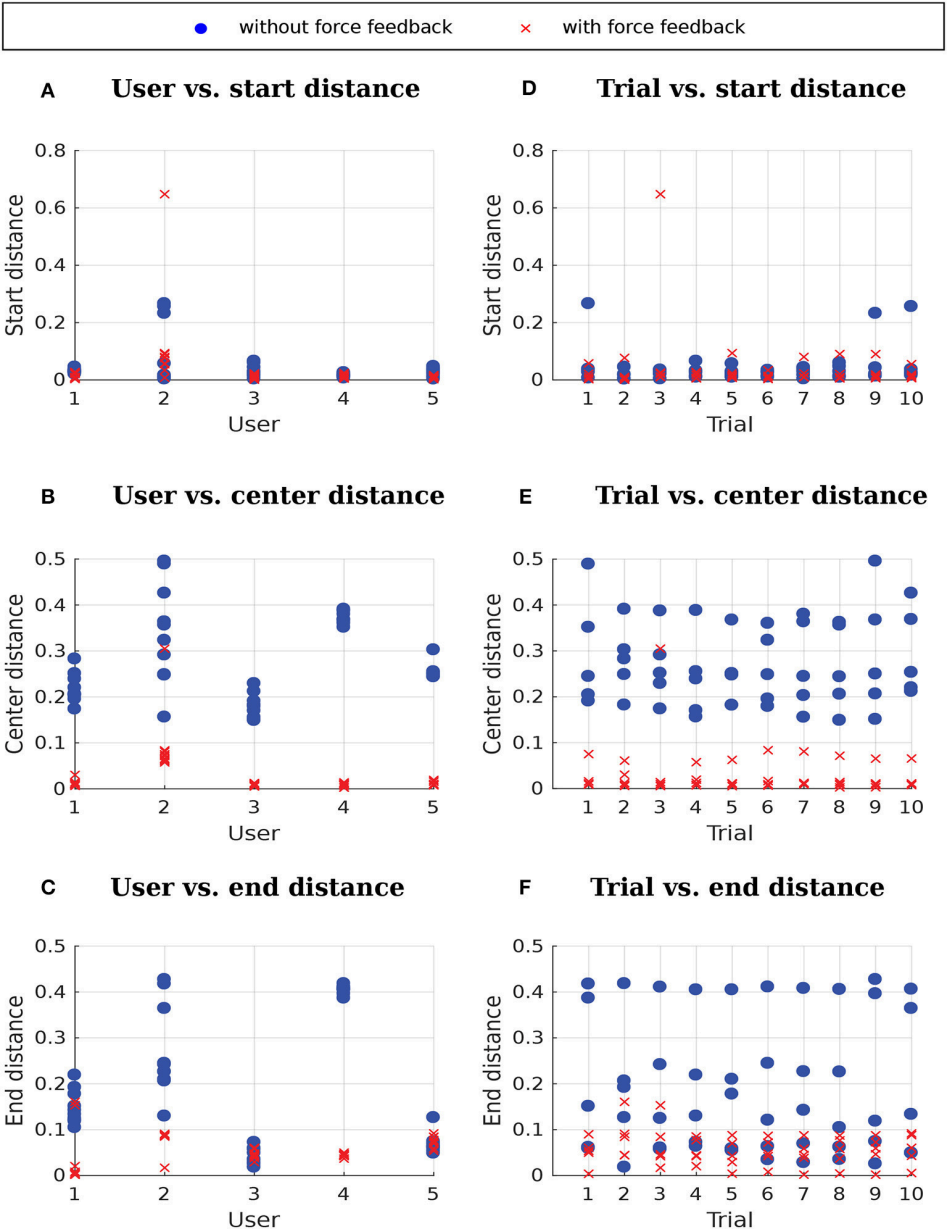
Distances without force feedback and with force feedback. **(A–C)** User vs. distances, where each data point corresponds to a different trial. **(D–F)** Trial vs. distances, where each data point corresponds to a different user.

Results showing the performance of the users with and without force feedback are presented in Figure 15. The use of force feedback did not greatly influence the distance to the start because the force feedback was activated only when the user pressed a button, right before starting to move. The start distance of the third trial of user 2 with force feedback is an outlier. This outlier was due to the user starting far away from the start position. The use of force feedback decreased the distance to the center of the window for all users and the distance to the end for three out of five users. The plots of trial vs. distances indicate that the users did not achieve a better performance with the force feedback only due to training through repetition because there is a clear difference between the performance in trials with force feedback and the performance in trials without force feedback.

A 2 (feedback) × 3 (distance measures) repeated-measures ANOVA was conducted to test the performance differences between the conditions with and without force feedback. The results reveal significant main effects of feedback [*F*_(1, 4)_ = 16.31; *p* < 0.05; $\eta_{p}^{2}=0.80$] and distance measure [*F*_(1, 5)_ = 12.93; *p* < 0.05; $\eta_{p}^{2}=0.76$; after *ϵ* correction for lack of sphericity] as well as a significant interaction of feedback × distance measure [*F*_(1, 5)_ = 10.10; *p* < 0.05; $\eta_{p}^{2}=0.72$; after *ϵ* correction for lack of sphericity]. Follow-up one-factor (feedback) repeated-measures ANOVA revealed a significant difference for distance to the center [*F*_(1, 4)_ = 57.32; *p* < 0.05; $\eta_{p}^{2}=0.94$], but not for distance to the start [*F*_(1, 4)_ = 0.11; *p* = 0.76] and end [*F*_(1, 4)_ = 3.61; *p* = 0.13], respectively. Therefore, feedback had only a significant and strong effect on the distance to the center. However, due to the small sample, the distance to the end test was slightly underpowered (1 − *β* = 0.798; corresponding to $\eta_{p}^{2}=0.474$). Thus, we conclude that force feedback has a differential influence on performance. Whereas force feedback does not influence initial error, later errors are expected to be substantially influenced by force feedback. However, further studies with bigger samples are required to confirm this conclusion.

As it can be seen in Figure 15C, users 3 and 5 were able to reach the desired end position with approximately the same accuracy with and without force feedback. Moreover, it has not been enforced in our experiments that users really finish their trials at the end position. Users have been instructed to finish their trials both with and without force feedback whenever they thought they have reached the end position. We could instead, for the trials with force feedback, instruct users to stop only when they feel force feedback contrary to the continuation of the trajectory, which could potentially help minimizing the variance of the end distance with force feedback as observed for users 1 and 2.

## 7. Conclusion and future work

This paper presents a probabilistic approach for assisting the practice and the execution of motor tasks by humans. The method here presented addresses the alignment in space and time of trajectories representing different executions of a motor task, possibly composed of multiple strokes. Moreover, it addresses building a probability distribution over demonstrations provided by an expert, which can then be used to assess a new execution of a motor task by a user. When no expert demonstrations are available, our system uses a novel reinforcement learning algorithm to learn suitable distributions over trajectories given performance criteria. This novel algorithm, named Relevance Weighted Policy Optimization, is able to solve optimization problems with multiple objectives by introducing the concept of relevance functions of the policy parameters. The relevance functions determine how the policy parameters are sampled when optimizing the policy for each objective.

We evaluated our framework for providing visual feedback to a user practicing the writing of Japanese characters using a computer mouse. Moreover, we demonstrated how our framework can provide force feedback to a user, guiding him/her toward correct movements in a teleoperation task involving a haptic device and a 3D environment.

Our algorithm to give visual feedback to the user practicing Japanese characters has still some limitations that could possibly be addressed by introducing a few heuristics. For example, the current algorithm assumes that the orientation of the characters is approximately the same. A correct character written in a different orientation would be deemed wrong by our algorithm. Procrustes Analysis (Goodall, 1991) provides a solution to align objects with different orientations. Our algorithm could be extended in the future with a similar technique to give meaningful feedback to the user regardless the orientation of the characters.

In our system, the user has to enter the correct number of strokes to receive feedback. For example, if the user is practicing a character composed of three strokes, the system waits until the user has drawn three strokes. Furthermore, the user has to write the strokes in the right order to get meaningful feedback, otherwise, strokes that do not really correspond to each other are compared. These limitations can potentially be addressed by analyzing multiple possible alignments and multiple possible stroke orders, giving feedback to the user according to the alignment and order that result in the best score.

Our current framework can give the user feedback concerning the shape of a movement, but not concerning its speed. In previous work (Ewerton et al., 2016), we have demonstrated how to learn distributions over shape and phase parameters to represent multiple trajectories with multiple speed profiles. Instead of giving the user force feedback toward the closest position along the mean trajectory, the distribution over phase parameters could be used to determine the speed of the attractor along the mean trajectory and how much the user is allowed to deviate from that speed. This extension shall be made in future work.

The framework proposed here could be applied in a scenario where a human would hold a brush connected to a robot arm. The robot could give the user force feedback to help him/her learn both the position and the orientation of the brush when writing calligraphy.

Especially if our framework can be extended to give feedback to the user concerning the right speed of a movement, it could potentially be applied in sports. This work could, for example, help users perform correct movements in weight training, such as in Parisi et al. (2016) and Kowsar et al. (2016). Another possibility would be to help users train golf swings given expert demonstrations or given optimized probability distributions over swings. The training of golf swings could be based on haptic guidance and use a similar setup as in Kümmel et al. (2014).

Future work should also explore further applications of the proposed Relevance Weighted Policy Optimization algorithm. In particular, it should be verified whether this algorithm can help finding solutions in more complicated teleoperation scenarios with different performance criteria, favoring, for example, smooth movements.

## Author contributions

ME contributed to this work by writing the manuscript, developing the proposed methods, coding, planning, preparing and executing the experiments. DR and JWe contributed to the code and to the preparation of the user experiments. JWi conducted the statistical analysis of our user experiments and contributed to the writing of the manuscript. GK, JP, and GM contributed to the writing of the manuscript.

### Conflict of interest statement

The authors declare that the research was conducted in the absence of any commercial or financial relationships that could be construed as a potential conflict of interest.
